# Successful treatment of Giant keloid on the right toes using trepanation combined with superficial radiotherapy (SRT-100): a case report with literature review

**DOI:** 10.3389/fmed.2024.1369953

**Published:** 2024-05-27

**Authors:** Ying-hua Song, Hui-min Zhu, Dan Chen, Zi-lu Qu, Liang Zhang, Li Wei

**Affiliations:** ^1^Department of Dermatology, Wuhan No. 1 Hospital, Wuhan, Hubei, China; ^2^Hubei Province and Key Laboratory of Skin Infection and Immunity, Wuhan No. 1 Hospital, Wuhan, Hubei, China; ^3^Wuhan No. 1 Hospital, Wuhan, Hubei, China

**Keywords:** keloids, superficial radiotherapy, trepanation, surgery, surgical excision

## Abstract

In dermatology, a keloid is one of the most common skin morphological abnormalities caused by excessive proliferation of fibroblasts. Keloids that are large or occur near important joint sites often cause varying degrees of physiological dysfunction in patients, therefore requiring medical treatment. A boy with congenital syndactyly developed huge keloids at the surgical site after undergoing surgical correction treatment. After treatment using trepanation combined with superficial radiotherapy (SRT-100) in our hospital, most of the boy’s keloids shrank and flattened. The affected foot returned to its normal appearance, and the boy could wear shoes normally. The boy did not complain of pain, numbness, or any other distinctive discomfort after completing the treatment. This suggested that the combination of trepanation and SRT-100 may be one of the options for treating hypertrophic keloids that cannot be treated by surgical excision.

## Introduction

Keloids are a benign fibroproliferative disorder that impairs the quality of life of patients by causing cosmetic disfigurement, pain, and pruritus (1, 2). Keloids can occur in any part of the human body, especially in the lower jaw, chest, back, and frequently moving joint areas (3). Hypertrophic keloids affect the normal appearance of the human body and limit normal joint function. Radiotherapy has a long history of application in the treatment of keloids, which was first described in 1906 (4), mainly including surgical excision combined with local wound irradiation to reduce the formation of scars or direct irradiation of the surface of scar tissue to induce scar atrophy and resolution (5). Radiotherapy induces excessive proliferation of fibrous tissue to shrink and undergo apoptosis, ultimately causing the scar to flatten. However, the radiation generated during radiotherapy is harmful to the human body and has carcinogenic effects. Before the emergence of computers, people were unable to control the generation and scattering of radiation, thus limiting its widespread application because of its harmful effect on the human body (6). With the advent of computers in modern society, computers have been combined with radiation-generating devices to accurately control the intensity, depth, and range of X-ray generation, minimizing X-ray scattering and leakage as much as possible and, subsequently, minimizing radiation damage to the human body while ensuring the efficacy of the treatment.

## Case presentation

**Figure 1:**
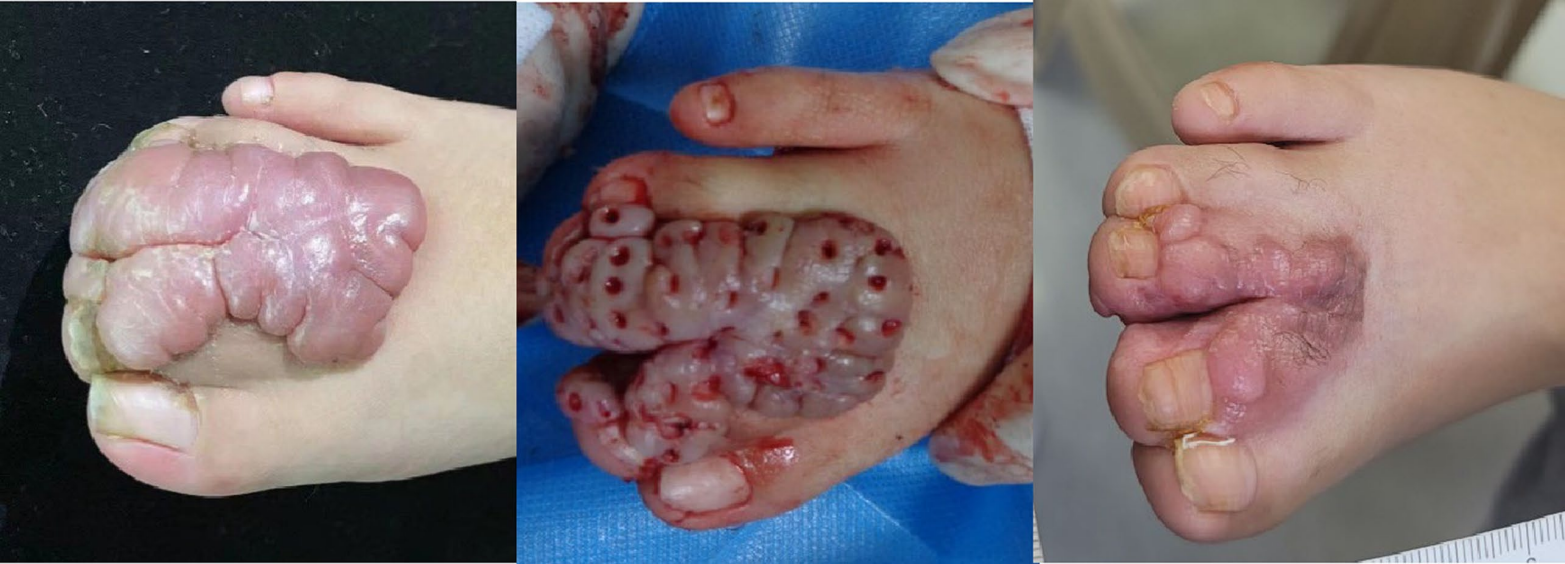
Before and after trepanation combined with superficial radiotherapy for giant keloid on the right toes of a boy. Left: Keloids located on the dorsal side of the second to fourth toes of the right foot. Middle: The boy was treated using trepanation on keloids under general anesthesia. Right: 6-month follow-up after the completion of superficial radiotherapy.

**Figure 2:**
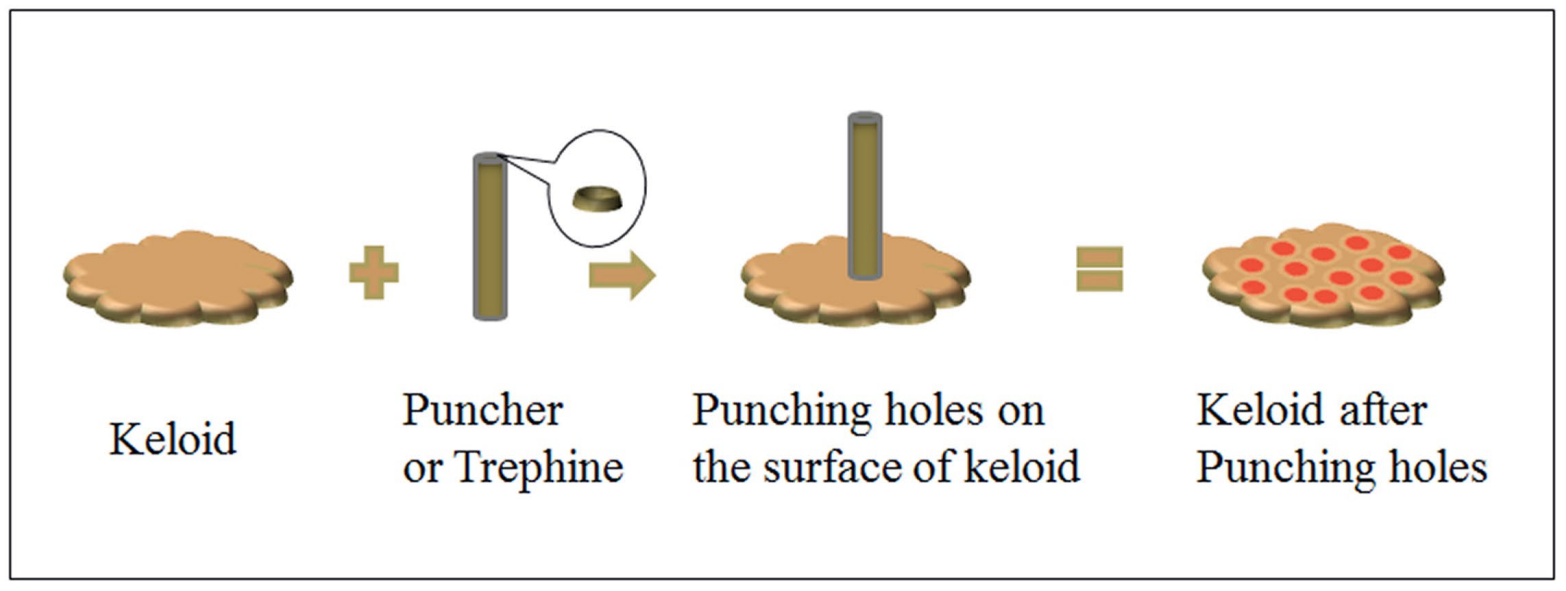
Schematic diagram of trepanation on the surface of keloid using a puncher.

**Figure 3:**
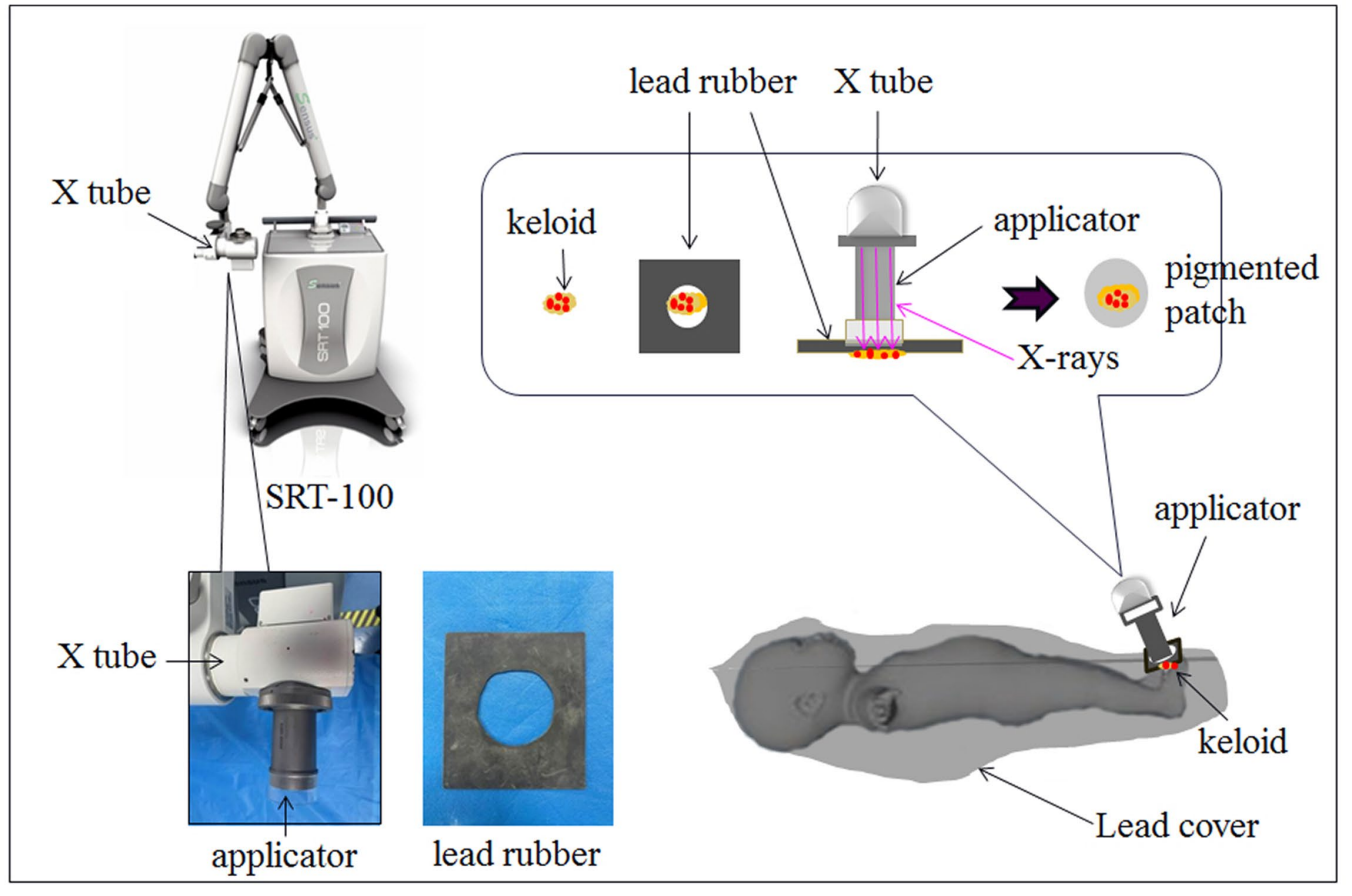
Schematic diagram of SRT-100 radiotherapy after trepanation for keloids. The non-irradiated area of the boy was tightly covered with lead clothing and lead rubber, leaving only the irradiated area exposed.

A 3-year-old boy had syndactyly of the toes of the right foot at birth. In September 2022, the boy underwent surgery to correct the syndactyly at a local hospital. One month after the surgery, the boy developed a soybean-sized lump at the surgical site, which gradually enlarged over time. His parents applied topical silicone gel to the lump for approximately 6 months, but the tumor did not shrink significantly. On physical examination, a giant hypertrophic proliferative plaque was observed on the dorsal side of the second to fourth toes of the boy’s right foot, with clear boundaries and a smooth and undamaged surface. He was treated using trepanation on keloids under general anesthesia and received the first superficial radiotherapy (SRT-100) 1 day after the surgery (Figures 1, 2). The treatment voltage was 70 kV, with a first radiation dose of 4.0 Gy followed by a daily dose of 3.5 Gy continuous irradiation for 3 days, totaling 4 days with a total dose of 14.5 Gy. The boy was followed up for 6 months after treatment. Most of the keloids atrophied, and the affected toes returned to their normal appearance (Figure 1). The non-irradiated area of the boy was tightly covered with lead clothing and lead rubber, leaving only the irradiated area exposed (Figure 3).

The boy received the first SRT-100 (Sensus Healthcare, United States) 2 h after trepanation, followed by daily continuous irradiation for 3 days, totaling 4 days. During and after the implementation of radiotherapy, the boy did not complain of pain, itching, or any other discomfort in the irradiated area. At 0.5 months after the completion of radiotherapy, there were large areas of hyperpigmentation in the irradiated area, and the wound healed normally with scabs. Hyperpigmentation completely disappeared after 3 months, and the hypertrophic keloid on the dorsal of the toes had significantly shrunken and flattened.

## Discussion

Keloids are overgrowths of a scar with excessive fibroblast proliferation and matrix deposition in the dermis, which can spread beyond the original boundary of the wound (7). Hypertrophic scars located in exposed areas of the body, such as the face, neck, or hands, can affect the normal appearance of the skin. Hypertrophic keloids located at the joint site affect normal joint activity; therefore, active intervention and treatment are required (8). There is almost no treatment that is considered completely effective in treating keloids. Systemic administration or local injection of corticosteroids, surgical excision combined with radiotherapy, silicone sheeting, liquid nitrogen cryotherapy, laser therapy, and photodynamic therapy have all been extensively used to treat this stubborn disorder (9, 10). The treatment outcomes vary in efficacy, and after the scar is healed, keloids easily recur. Among the numerous therapeutic options, combining surgical interventions with radiation has been favorably advocated, having a high cure rate and a low recurrence rate. In comparing radiation modalities, postoperative brachytherapy had the lowest recurrence rate of 15%, compared with a 23% recurrence rate for X-rays and a 23% recurrence rate for electron beam radiation (11). Studies have reported that excision followed by radiation is safe and practical (11, 12). Other options may be supplements to this plan, such as local injections of corticosteroids. Some patients may have special contraindications, such as keloids located near the eyes or thyroid, that are not suitable for radiotherapy, and they have to choose some second-best treatment plans, such as liquid nitrogen cryotherapy and laser therapy.

Some patients may have hypertrophic keloids caused by surgical incision, which may not be respectable due to factors such as location; for example, keloids located in the distal part of limbs with less skin tissue and high tension. Systemic administration and local injection of corticosteroids have little therapeutic effect, and laser therapy is less effective in removing hypertrophic scar tissue. Moreover, stimulation of the proliferative area through laser therapy will further cause scar tissue to thicken. Liquid nitrogen cryotherapy does not have a significant therapeutic advantage for hypertrophic scars either. Photodynamic therapy exhibits good effects, but the treatment process is long, and most patients experience obvious pain and some other discomforts. Therefore, non-cooperative pediatric patients are not suitable for this treatment. Furthermore, it is still debatable whether photodynamic therapy is effective in treating hypertrophic keloids (13). We performed trepanation combined with SRT-100 on this male patient, and the results confirmed the good effect of this treatment.

Numerous literature studies have revealed that the consensus on the best treatment for keloids is the combination of surgical therapies and radiotherapy, such as brachytherapy (14), which has been proven to be safe and effective and has a low recurrence rate. The treatment plans vary worldwide, among which the currently recognized treatment plan is 20 Gy in five fractions. Because of the small amount of skin tissue in the affected area and the high local tension, the trepanation we used in this treatment was a partial scar excision, and within 24 h after the surgical treatment, the patient received the first SRT-100. We performed the succeeding treatment once a day for three consecutive days, with a total of four fractions of SRT-100. We reviewed radiotherapy plans for keloids combined with surgical incision, with radiation treatment ranging from one to five fractions and radiation doses ranging from 2 to 10 Gy per fraction, for a total dosage of 8 to 20 Gy. The voltage is generally 50–70 kV. The efficacy varies, but there are no obvious adverse reactions (15). We comprehensively evaluated the degree of hypertrophic scar tissue in this male patient and referred to the literature on safe doses for radiotherapy (16). We finally developed a plan of four fractions with a voltage of 70 kV and a total dose of 14.5 Gy for the treatment of this male patient.

Superficial X-rays are preferable because of their ease in field shaping and superior protection from scatter radiation from sensitive nearby structures (17). Therefore, they can be safely used to irradiate diseased areas near important organs. The SRT-100 is a recently developed mobile superficial therapy system by Sensus Healthcare. This X-ray generation equipment, controlled by computers, can accurately control the X-ray energy, irradiation range, irradiation time, and irradiation depth. SRT-100 delivers dose rates comparable with those of electrons and provides several safety features to meet modern requirements (18). The boy’s scar was located in a thin and high-tension area of the skin tissue on the back of the right toes, so it could not be completely surgically removed. The hypertrophic scar severely affected the motor function of the affected foot after the syndactyly correction surgery, and the deformed appearance also had a psychological effect on the child. We attempted to adopt scar trepanation technology as a secondary approach to maximize the preservation of the patient’s limb physiological function while partially removing the hypertrophic scar. Immediate postoperative adjuvant therapy with SRT-100 proved to be highly effective. After treatment, the vast majority of hypertrophic scar tissue shrank and flattened, the boy’s foot returned to its appearance before the deformity correction surgery, and the joint function of the foot was also partially restored. However, a noticeable protruding scar at the third toe of the right foot remained, which requires readmission for secondary trepanation combined with SRT-100 in the future. Because the boy’s father refused any further surgery, the child’s foot deformity was ultimately not corrected.

The combination of trepanation and SRT-100 may be one of the options for treating hypertrophic keloids that cannot be treated by surgical excision or for patients who cannot undergo surgical treatment. However, as a radiotherapy option, SRT-100 has inevitable radiation damage, so pregnant or lactating women are not suitable for this treatment. In addition, there are strict restrictions on the safe cumulative dose of radiotherapy, so multiple courses of treatment cannot be repeated. Excessive radiation can increase the incidence of various malignant tumors, which is the limitation of this treatment strategy. Furthermore, as keloids are prone to recurrence, we only had a follow-up time of 6 months after treatment for the patient. Therefore, it is vital to extend the follow-up time for a more comprehensive evaluation of treatment efficacy.

## Ethics statement

Written informed consent was obtained from the participant/patient(s) for the publication of this case report.

## Author contributions

Y-hS: Writing – review & editing, Writing – original draft. H-mZ: Writing – review & editing, Data curation. DC: Writing – review & editing, Resources. Z-lQ: Writing – review & editing, Methodology, Formal analysis. LZ: Writing – review & editing, Supervision, Funding acquisition. LW: Writing – review & editing, Supervision, Project administration, Funding acquisition.
